# Application of functional genomics to primate endometrium: insights into biological processes

**DOI:** 10.1186/1477-7827-4-S1-S4

**Published:** 2006-10-09

**Authors:** Linda C Giudice

**Affiliations:** 1 Department of Obstetrics, Gynecology and Reproductive Sciences, University of California, San Francisco, 505 Parnassus, M1496, Box 0132, San Francisco, CA 94143-0132, USA

## Abstract

Endometrium is a dynamic tissue that responds on a cyclic basis to circulating levels of the ovarian-derived steroid hormones, estradiol and progesterone. Functional genomics has enabled a global approach to understanding gene regulation in whole endometrial tissue in the setting of a changing hormonal milieu. The proliferative phase of the cycle, under the influence of estradiol, has a preponderance of genes involved in DNA synthesis and cell cycle regulation. Interestingly, genes encoding ion channels and cell adhesion, as well as angiogenic factors, are also highly regulated in this phase of the cycle. After the LH surge, different gene expression profiles are uniquely observed in the early secretory, mid-secretory (window of implantation), and late secretory phases. The early secretory phase is notable for up-regulation of multiple genes and gene families involved in cellular metabolism, steroid hormone metabolism, as well as some secreted glycoproteins. The mid-secretory phase is characterized by multiple biological processes, including up-regulation of genes encoding secreted glycoproteins, immune response genes with a focus on innate immunity, and genes involved in detoxification mechanisms. In the late secretory phase, as the tissue prepares for desquamation, there is a marked up-regulation of an inflammatory response, along with matrix degrading enzymes, and genes involved in hemostasis, among others. This monograph reviews hormonal regulation of gene expression in this tissue and the molecular events occurring therein throughout the cycle derived from functional genomics analysis. It also highlights challenges encountered in using human endometrial tissue in translational research in this context.

## Background

**Figure 1:**
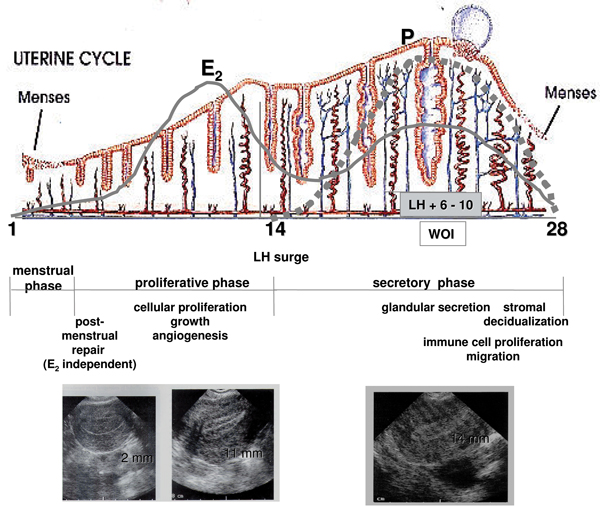
**Cyclic changes in endometrium across the cycle**. Shown are hormonal profiles, cycle phases and sub-stages, and ultrasonographic appearance of the endometrium in early proliferative, late proliferative, and mid-secretory phases. Reproduced with permission of Adeza Biomedical.

Human endometrium, the anatomic prerequisite for continuation of the species, is a dynamic tissue that responds to the circulating steroid hormones, estradiol (E_2_) and progesterone (P), throughout normal menstrual cycles (Figure 1; and [1], review). The goals of orchestrated events in endometrium are to permit successful nidation of a conceptus, and in the absence of such, desquamation of the tissue and subsequent regeneration. While the window of implantation is a temporally and spatially defined time in the endometrial cycle in which blastocyst implantation can begin, events prior to it are critical in optimizing endometrial receptivity to embryonic implantation in this time frame. In the absence of implantation, orderly shedding of the tissue and hemostasis are essential to prevent morbidities of hypermenorrhea, menorrhagia and anemia ([1], review).

There are numerous approaches to assess endometrial function, including (a) direct observation through, e.g., hysteroscopy or gross examination of the tissue; (b) imaging techniques, including ultrasound and magnetic resonance imaging; and (c) endometrial biopsy or hysterectomy specimen tissue sections and (i) subsequent hematoxylin and eosin staining and/or immunohistochemical or in situ hybridization analysis for specific gene products and/or (ii) simultaneous analysis of multiple genes and gene products by array analyses for gene expression and proteins. The latter approaches (ii) have been relatively recent events, in contrast to histologic evaluation of cycle-dependent changes in endometrium, first described over half a century ago [2]. It should be noted that histology has been the classical approach for evaluation of adequacy of the endometrium for fertility and normalcy of the endometrium in clinical conditions of abnormal uterine bleeding, where, e.g., polyps, fibroids, and endometrial hyperplasia and/or cancer have been suspected. This type of evaluation is standard of care in the clinical setting where abnormal uterine bleeding or unusual findings on ultrasound or at hysteroscopy and hysterectomy have been made. However, recent studies have called into question the utility of the endometrial biopsy as a clinical tool for fertility evaluation and for research because there is significant inter- and intra-observer variability and histologic delay fails to discriminate between fertile and infertile couples [3]. Also, Murray and colleagues have demonstrated that histologic features fail to distinguish reliably specific menstrual cycle days or narrow intervals of days [4]. Furthermore, histology rarely gives insight into the molecular mechanisms occurring in the tissue throughout the cycle, which may be accomplished through molecular phenotyping. Questions that arise about molecular phenotyping of human endometrium include whether this approach can (a) distinguish among the phases of the cycle; (b) define receptivity to embryonic implantation; (c) identify a variety of endometrial disorders not apparent from histologic evaluation of the tissue; and (d) give insight into molecular events that occur dynamically throughout the cycle.

The genomic era has heralded a new approach for simultaneous analysis of genes and proteins in tissues, and this monograph describes gene expression studies performed on whole endometrial tissue in women and non-human primates. The major limitation of such studies is that gene transcription alone has been investigated in the absence of investigation into the corresponding proteome. The latter would not only confirm gene expression profiling, but also would give insight into proteins and protein modifications that participate in biological processes during the endometrial cycle. Furthermore, a proteomic approach would be welcome to detect changes in secreted proteins and soluble endometrial biomarkers across the cycle and in endometrial disorders. While elucidation of the genome has been an extraordinary accomplishment during the past decade, elucidation of the proteome, technologically even more challenging and expensive, must be conducted for a more accurate assessment of endometrial physiology and pathophysiology.

### Challenges in Using Human Tissues

The endometrium is comprised of zones that include the functionalis (shed on a monthly basis) and the basalis (believed to be the origin of cells for regeneration of the tissue) ([1], review). These zones are clearly identifiable by the naked eye in hysterectomy specimens, although they are not separated by an anatomic barrier (e.g., membrane), and thus tissue sampling often includes some cell populations from each layer. This is particularly true with currettings from hysterectomy specimens. Less contamination from basalis endometrium is encountered in endometrial biopsies, where the functionalis is the primary layer sampled. In addition, biopsies or selecting only a portion of tissue in hysterectomy specimens may result in different complements of cell types in different specimens analyzed. This can lead to significant variability in genes expressed in tissue biopsies during the same cycle phase and in specimens compared in different phases across the cycle. These issues are important to consider in experiments designed to evaluate whole tissue genome or proteome expression.

Obtaining normal human tissue, and in particular, normal hormone-dependent tissues from cycling women, for biochemical analysis is a challenge and underscores some important issues associated with this type of translational research. Endometrial specimens should be obtained from the fundus, and the operator should be careful to avoid non-hormone-dependent parts of the endometrium, such as the periostia. Furthermore, accurate subject history annotation is important, as steroid hormones have significant effects on endometrial function and gene expression, and excluding recent exogenous steroid hormone use is essential. Even in this setting, obtaining sufficient tissue for analysis can sometimes be a challenge, as well as excessive blood in specimens to be analyzed.

Perhaps one of the most challenging issues, however, is how to define normal endometrial tissue. Most samples are obtained from subjects undergoing hysterosocopy and/or endometrial biopsy or hysterectomy for clinical indications such as infertility, abnormal uterine bleeding, uterine fibroids, uterine prolapse, pelvic pain, and endometriosis. It is rare to obtain endometrial tissue from normal cycling subjects not on contraceptive steroids and willing to donate their endometrium to science. Thus, subject selection and carefully documented and validated medical and surgical histories are critical to obtaining data that are reproducible and meaningful.

### Endometrial gene profiling

**Figure 2:**
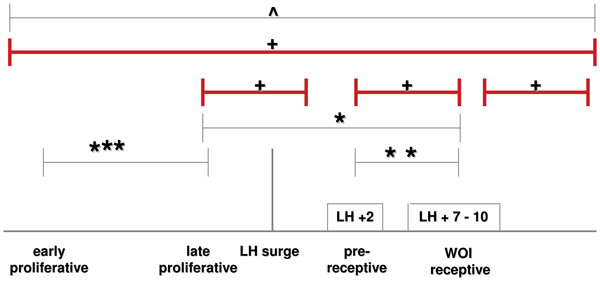
**Schematic representation of microarray studies comparing cycle phases in women and non-human primates**. (See text). Symbols correspond to citations: * [5,7,9]; ** [6,8,11,12]; *** [10]; ^[13]; + [14]. With permission from reference [14].

Several investigators have recently reported results of cDNA and oligonucleotide array analyses of endometrial gene expression in humans and non-human primates. Figure 2 summarizes these studies with regard to phases of the cycle analyzed, primarily for pair wise analyses [5-12], although two studies investigated gene expression across the cycle [13,14]. Herein, we focus on the global gene expression profiling of human endometrium across the menstrual cycle and select pair-wise comparisons of phases in the studies of Talbi, Hamilton et al [14] and Ponnampalan et al [13], which used whole genome 54,600 genes and ESTs on high density oligonucleotide microarrays and 13, 600 cDNA arrays, respectively.

**Figure 3:**
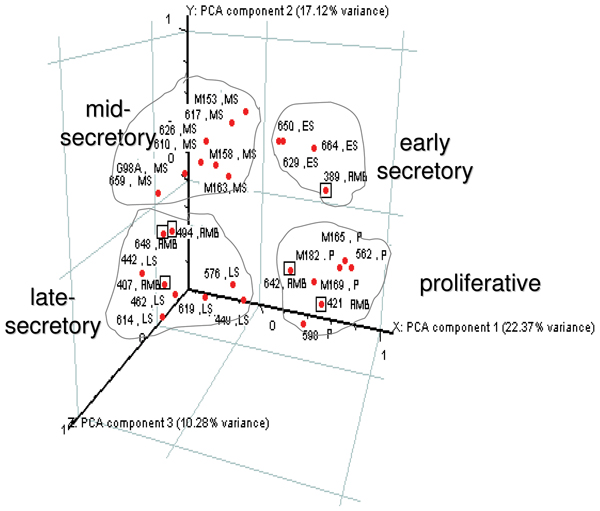
**Gene clustering in the endometrium: Principal component analysis (PCA) of human endometrium across the menstrual cycle**. PCA was applied to all endometrial samples, and numbers refer to individual sample labels. "Ambiguous samples" (see text) are labeled AMB (dots in squares). Analysis reveals clustering of samples into cycle phases.With permission from reference [14].

**Figure 4:**
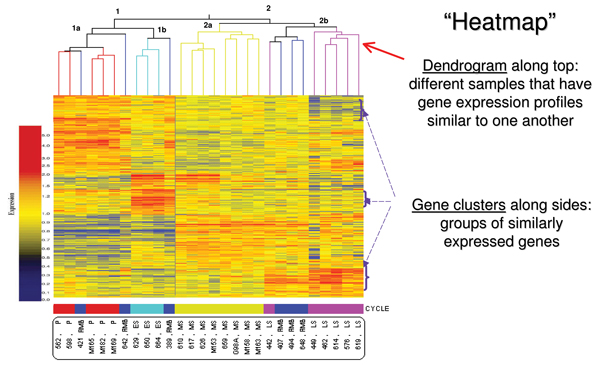
**Gene clustering in the endometrium: Gene expression profiling across the menstrual cycle**. The Heatmap represents relative gene expression levels in the endometrial samples across the menstrual cycle. Each horizontal line represents a single gene, and each column represents a single sample. Samples cluster by cycle phase (bar at bottom of the Heatmap: proliferative (red), early-secretory (light blue), mid-secretory (olive), late secretory (purple) and ambiguous samples (dark blue). Relative expression of each gene is color-coded: high (red) or low (blue), as indicated in the color legend on the left side of the figure. Hierarchical clustering reveals sample clustering into cycle phases and unique molecular profiles. Adapted from [14], with permission.

In the study by Talbi, Hamilton, et al [14], 46 subjects were recruited, but only 22 had endometrial samples that yielded sufficient and good quality RNA for subsequent microarray analysis. Six samples either had different histologic readings from two or more pathologists or had a histologic evaluation by only one pathologist and were designated as ''ambiguous''. This study provided the first, whole genome-wide gene, gene ontology, and gene clustering analyses across the entire menstrual cycle in normo-ovulatory women. Different data analyses were conducted to determine how samples clustered together (Figures 3 and 4). A completely unbiased approach, principal component analysis (PCA, Figure 3), used the entire gene set (54,600 probe sets); whereas hierarchical clustering analysis (Figure 4) used a more limited gene set (7,231 probe sets from pair-wise comparisons of early secretory (ESE) vs. proliferative (PE), mid-secretory (MSE) vs. ESE, and late-secretory (LSE) vs. MSE)). These analyses demonstrate several important points: (a) PCA analysis reveals that samples with known histology cluster into cycle phases; (b) hierarchical clustering reveals that samples with known histology cluster into the same cycle phases as observed with the PCA analysis; (c) hierarchical clustering analysis reveals two major branches and several sub-branches, with PE and ESE clustering together in the first major branch and MSE and LSE in the second major branch (Figure 4); (d) hierarchical clustering reveals that samples have unique molecular profiles; (e) samples with unknown or ''ambiguous'' histology cluster into cycle phases that are the same by both PCA and hierarchical clustering analysis; (f) samples cluster independently of how they were obtained (biopsy or curetting); and (g) samples cluster independent of the clinical indication for which the ''normal'' specimen was obtained. Thus, this study demonstrates that assignment of menstrual cycle stage of ambiguous samples, based on their gene expression profiles and their cluster grouping, is a powerful adjunct to the historical histological ''gold standard'' of endometrial assessment. Furthermore, it demonstrates that samples obtained at any time in the cycle have unique molecular signatures that preclude the need to assign a histologic phase to the sample *a priori*. While unique gene signatures lead to clustering in groups (phases), it is interesting that the molecular signatures are not identical (Figure 4) and may be the result of, e.g., subject-to-subject variability and different complements of cell types in different specimens.

The study of Ponnampalan et al [13] used cDNA microarrays to study endometrial gene expression across the menstrual cycle. The investigators performed initial hierarchical clustering analysis of 43 endometrial samples that revealed two main branches: one containing menstrual (M), early (EPE)-mid (MPE) proliferative, late proliferative (LPE), and ESE-MSE, and the second containing M, M/EPE, MSE, LSE, and LSE/M. Six outliers were subsequently removed for disagreement of histologic stage between two pathologists or for poor hybridization, and this resulted in two main branches for 37 samples. One branch contained M, EPE-MPE, LPE-ESE, and ESE-MSE and the other contained M, MSE-LSE, and LSE-M. Some cycle phases had too few samples for analysis, and these (MSE and LSE) were then merged. Thus, it is difficult to compare gene directly expression profiles between these two studies, due to these different approaches for data analysis, as well as different platforms for hybridization.

### Early Secretory vs. Proliferative Endometrium

During the proliferative phase, endometrium is stimulated by high levels of circulating E_2_, and then after ovulation in the early secretory phase, it is the target of low, but rising, circulating levels of P (and E_2_). Thus, genes regulated in ESE vs. PE may be regulated by E_2 _and/or P. With regard to E_2_-regulated genes, insight into these derive from a recent study on gene expression in human endometrium in late PE (LPE, high E_2_) compared to menstrual endometrium (very low E_2 _levels) and E_2_-treated endometrial explants, using the same Affymetrix platform [10]. Genes up-regulated in LPE vs. menstrual endometrium included oviductal glycoprotein-1, connexin-37, olfactomedin-1, SFRP4; and down-regulated genes included MMPs-1, -3, and -10, IL-1b, IL-8, -11, inhibin bA, SOX4 [10]. In the study by Talbi, Hamilton et al [14], olfactomedin-1 was down-regulated in ESE, suggesting that P inhibits its expression in this phase of the cycle. SOX4 was down regulated in LP vs. menstrual endometrium [10], and it is also down-regulated in ESE vs. PE [14], suggesting that E_2 _down-regulates this gene. A recent study on global gene expression (12,000 genes/ESTs) demonstrated that E_2 _treatment of human endometrial cells resulted in up-regulation of N-cadherin [15]. However, N-cadherin was down-regulated in ESE vs. PE [14], suggesting that N-cadherin expression is inhibited by P. Of interest, also, is the up-regulation of FOXO1A (2.1-fold) in ESE vs. PE [14], especially in view of recent data in breast cancer cells that demonstrate the importance of FOXO1A in E_2 _action [16].

In a recent study by Tan et al [17], global gene profiling of mouse uterus during the estrous cycle was investigated and revealed up-regulation of 17bHSD-2 in estrus versus diestrus. This gene is the highest up-regulated gene in ESE vs. PE in human endometrium[14]. Although the data in mouse uterus suggest that it is E_2_-regulated, data are convincing that 17bHSD-2 in human endometrium is regulated via PR in the stroma, with paracrine factors up-regulating it in the epithelium [18]. Up-regulation of 17bHSD-2 in human ESE vs. PE is also consistent with results from Mustonen et al [19]. The up-regulation of 17bHSD2 suggests that direct E_2 _action in this phase is beginning to be curtailed, as a prelude to more pronounced curtailment in the mid-secretory phase in which estrogen receptors are down-regulated in the stroma and nearly completely absent in the epithelium [20-23].

Several genes are up-regulated in ESE vs. PE that are worthy of mention, including MUC-1, Dkk-1 and other Wnt family members, and IL-15 [14]. MUC-1 maintains hydration of cell surfaces, lubricates them, and protects from microorganisms and degradative enzymes [24]. In mouse uterus it is stimulated by E_2_, but it is highly up-regulated in the secretory phase in human endometrium and is presumably P-regulated [24]. Dkk-1 is up-regulated > 6-fold in ESE vs. PE, and recent data from our laboratory demonstrate that it is a P-regulated gene in endometrial stromal cells [25]. IL-15 is P-regulated and is important as a chemoattractant and stimulator of NK cell replication [26,27].

ESE is characterized by inhibition of cellular mitosis, in marked contrast to the mitotic activity that occurs in PE, and the down-regulation of numerous growth factors in this phase further supports this conclusion [14]. Furthermore, the shift to cellular metabolism in this phase of the cycle, compared to PE, underscores that ESE is biosynthetically active, likely in preparation for embryonic implantation.

### Mid Secretory vs. Early Secretory Endometrium

The mid-secretory phase is replete with numerous biological processes and molecular participants that coordinately facilitate embryonic implantation. This is the most well characterized phase of the cycle, with regard to gene expression analysis [6,8,11,14]. Despite the fact that these studies all used similar platforms, the number of genes in common among ALL studies in different gene ontology categories is surprisingly low. Of the 75 genes up-regulated in MSE vs. ESE in the study by Riesewijk et al [8], 41 are identical to up-regulated genes in the study by Talbi, Hamilton et al [14], and of the 56 down-regulated genes, 11 were the same. Of the 74 genes encoding cell surface components, extracellular matrix components, growth factors, and cytokines in MSE vs. ESE in the study by Carson et al [6], 11 were in common, and of the 76 down-regulated genes, only one was in common with Talbi, Hamilton et al [14]. Of the 49 up-regulated genes in MSE vs. ESE in the study by Mirkin et al [11], 14 are similarly regulated, and of the 58 that were down-regulated, only 3 are common to Talbi, Hamilton et al [14]. These differences likely are due to multiple factors, including different chip versions, different hybridization conditions, scanners, and statistical programs for data analyses, subject-to-subject variability, where in endometrium samples were obtained (fundus, lower uterine segment, periostium), different complements of cellular components in individual samples, and precisely the time in the cycle when samples were obtained. With regard to the latter, e.g., Reisewick et al [8]) obtained endometrial tissue strictly at 2 and 7 days after the LH surge; whereas, most other studies have samples that span groups of days in a particular phase.

#### Up-Regulated Genes

Gene families that were up-regulated in MSE vs. ESE are relevant to the cellular differentiation and cell-cell communications that underlie receptivity to embryonic implantation. These include the processes of cell adhesion, suppression of cell proliferation, regulation of proteolysis, metabolism, growth factor and cytokine binding and signaling, immune and inflammatory responses, and the responses to wounding and stress [14]. Striking up-regulation was observed with genes encoding secreted proteins, cytokines and genes involved in detoxification mechanisms. In the study of Ponnampalam et el [13], pair-wise comparison of genes expressed in MSE vs. ESE was not reported, and thus comparisons between the data sets is not possible.

##### Immune genes

The most-highly up-regulated gene (61-fold) in MSE vs. ESE is CXCL14, a chemokine that is also known as breast and kidney expressed chemokine (BRAK). It recruits monocytes in the setting of inflammation and without inflammation [28], and it may be a major recruiter of monocytes and other cell types to endometrium during the implantation window. Leukemia inhibitory factor (LIF) is significantly highly up-regulated in MSE vs. ESE [14,29]. It plays a central role in endometrial receptivity in the mouse [30,31], and increasing evidence suggests that it is also important in humans [32]. For example, in some women with infertility and repetitive miscarriage, low levels of LIF in MSE have been reported [33,34], as have point mutations in the coding region of the LIF gene [32]. The data set reveals, overall, an up-regulation of genes involved in activation of the innate immune response, including members of the complement family, antimicrobial peptides, and Toll-like receptor expression [14]. There is also enhancement of chemotaxis of monocytes, T cells, and NK cells by candidate genes CXCL14, granulysin, IL-15, carbohydrate sulfotransferase 2, and suppression of NK and T-cell activation. IL-15 is regulated by P (see above), and recent evidence suggests a central role for IL-15 in secretory phase endometrium in the recruitment of peripheral blood CD16-NK cells into the tissue in this phase of the cycle [35]. Many of the genes observed in our data set were also observed in the data from Carson et al [6], Riesewijk et al [8], and Mirkin et al [11]. The gene expression profiles are consistent with the marked increase in lymphocyte infiltration [2,36].

##### Secretory proteins

Other genes that were highly up-regulated include cysteine-rich secretory protein (CRISP) 3 and secretoglobin family 2A member 2, believed to be important in embryonic attachment, although other functions may exist in this time of the cycle.

##### Detoxification

Among the most highly up-regulated genes are those encoding glutathione peroxidase-3 (GPX-3) and metallothioneins (MT) 1G,1H,1E,1F,1L,1X, and 2A. The former is consistent with prior observations [8,11] (and in MSE vs. PE [5,7,37]). GPXs (anti-oxidants) and metallothioneins protect cells from unstable reactive radicals and heavy metals [38]. These are likely to protect an embryo from free radicals and heavy metals in the very beginning phases of implantation. Interestingly, GPXs are selenium-dependent, and women with selenium deficiency have a higher rate of infertility and miscarriage [39].

#### Down-regulated genes

Among the most highly down-regulated genes in MSE vs. ESE are secreted frizzled related protein (SFRP), olfactomedin 1, the progesterone receptor (PR), PR membrane component 1, ER-a, MUC-1, 17bHSD-2, and MMP-11 [14]. Many were not found to be regulated in earlier MSE vs. ESE microarray studies, likely due to reasons listed above for differences in up-regulated genes among various studies.

#### Progesterone regulated genes

**Figure 5:**
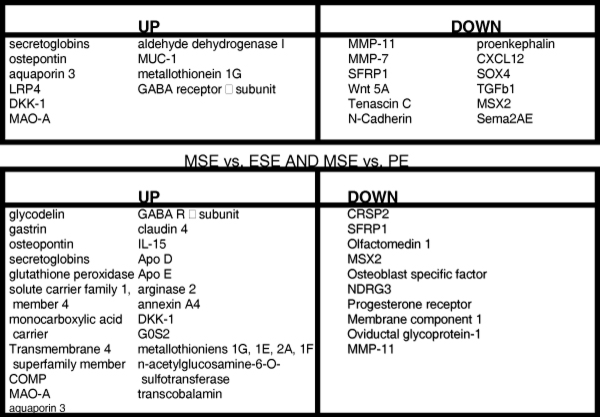
**Candidate progesterone-regulated genes in human endometrium**. Candidate genes were derived from genes common to the comparisons between early secretory endometrium (ESE) vs. proliferative endometrium (PE) and mid-secretory endometrium (MSE) vs. ESE (upper panel) and MSE vs. ESE and MSE vs. PE. With permission from (14).

Analysis of genes expressed in various phases of the menstrual cycle can give insight into genes that are candidates for regulation by P. Of interest is the regulation of genes in MSE vs. PE from *all *studies in humans and non-human primates [5,7,9] that are *in common *with those regulated in ESE vs. PE [14] and MSE vs. ESE [6,8,11,14]. The result of this comparison is shown in Figure 5. These genes are likely to be regulated by P, either directly or indirectly, although validation of such regulatory mechanisms awaits further investigation. Analysis of PREs and EREs by Borthwick et al [7] and Mirkin et al [11] support some of these conclusions, as do studies using anti-progestins and PR knock out of P-regulated genes in mouse uterus/endometrium [40].

### Late Secretory vs. Mid Secretory Endometrium

The transition from mid-secretory to late secretory endometrium, in the absence of embryonic implantation, is characterized by P withdrawal and preparation for desquamation of the tissue and menstruation. Accordingly, gene expression profiling reveals changes in genes involved in the extracellular matrix, the cytoskeleton, cell viability, vasoconstriction, smooth muscle contraction, hemostasis, and transition in the immune response to include an inflammatory response [14,41,42].

#### Extracellular matrix degradation

In the microarray data set for LSE vs. MSE, striking up-regulation of metalloproteinases (MMPs and ADAMs), serine proteases [the plasminogen activators, uPA (PLAU) and tPA (PLAT)], and their inhibitors, was observed [14]. This is consistent with declining P levels within the tissue at this time of the cycle and concomitant dysinhibition of expression of these genes. P also inhibits leukocyte transit into the endometrium [43], a process that is controlled in the secretory phase by the stromal cell, the main cell type that retains PR in this phase of the cycle [20-23]. uPA (PLAU) is up-regulated in LSE compared to MSE, and can activate TGFb1 [44]. It is regulated by tissue factor which is also P-dependent, and it activates plasminogen which can further activate MMPs [45]. Thus, it is likely that uPA plays a major role in preparing the tissue for desquamation. Another group of genes that are important in endometrial breakdown are members of the TGFb family. For example, endometrial bleeding associated factor (EBAF) is one of the most highly up-regulated genes in LSE vs. MSE [14]. EBAF stimulates expression proMMP-3 and -7 in proliferative phase human endometrial explants, and P inhibits this as well as inhibition of EBAF expression [46]. Importantly, these genes are up-regulated, but tissue breakdown does not occur. This programming of the endometrium in the late-secretory phase appears to be inhospitable for embryonic implantation and must be carefully regulated, temporally, for menses to occur at an appropriate threshold of gene activation, and to permit subsequent repair of the tissue for the next cycle.

#### Immune activity

The immune gene regulation observed in LSE vs. MSE [14] reflects known histologic observations of an influx of polymorphonuclear leukocytes [2,36]. Fc receptors, MHC molecules, NK molecules and T cell molecules are all up-regulated, and it appears that the system is preparing for immune action involving innate and adaptive immunity (T cell specific, and antibody-mediated). The profile of genes suggests a pro-inflammatory response, with up-regulation of IL-1, a pro-inflammatory cytokine that induces T cell activation. In addition, IL-b and TNF-a produced by leukocytes in the stromal compartment in the late secretory phase, stimulate release of matrix degrading enzymes that contribute to the breakdown of the vascular basement membrane and connective tissue integrity in the functionalis layer [47,48]. Also, numerous molecules in T cell signaling/activation are up-regulated [14]. In addition to IL-1b, other key inflammatory mediators, e.g., CXCL8 (IL-8), CCL2 (MCP-1) [42,49], and the synthesis of prostaglandins, by induction of COX-2 [49,50] are all up-regulated in response to withdrawal of P. The microarray data are consistent with a major shift from an innate immune response in MSE to an inflammatory response in LSE [51,52]. Such an inflammatory environment, along with up-regulation of matrix degrading enzymes and cellular apoptosis, teleologically would not be a receptive environment for embryonic implantation. Indeed, we have suggested that gene expression profiles in LSE vs. MSE may serve to define closure of the receptive period and the onset of the subsequent non-receptive period of endometrial development in normal, non-conception cycles. Of interest are the patterns of immune gene regulation in LSE in a conception cycle and the cross-talk between the implanting conceptus and the maternal decidua.

## Summary

**Figure 6:**
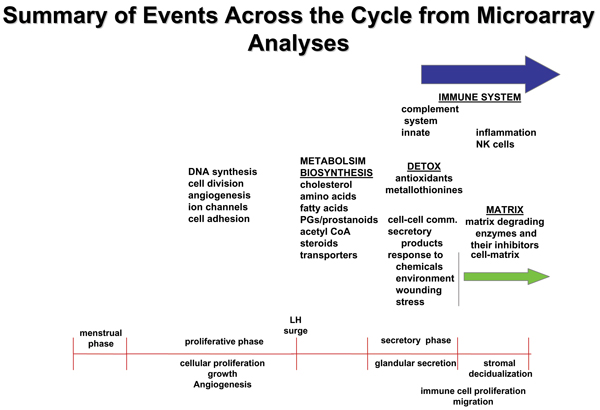
**Summary of events occurring across the menstrual cycle, deduced from microarray analyses**. (See text).

Microarray analyses have provided global inspection of the endometrium across the cycle and insight into the biological processes occurring therein. A schematic summary of this is presented in Figure 6. It is likely that there are microenvironments in which some processes are occurring more than others, as proximity to vascularity and immune cells may affect endometrial cellular function and vice-versa. Remarkably in normally cycling women, there is a regularity of cycle length that persists cycle-to-cycle, with efficient tissue desquamation and self-limited bleeding. Abnormalities in cycle length and receptivity to embryonic implantation, as well as endometrial bleeding disorders and other endometrial pathologies likely reflect significant changes in endometrial gene expression and (dys)regulation. Microarray analyses reviewed herein are but the beginning of a compendium of genes identified in understanding the dynamic changes in morphology and function of the endometrium in health and disease. These approaches also offer the opportunity to identify abnormalities in endometrium (e.g., endometrial hyperplasia, cancer, endometriosis, endometrial polyps, and in the setting of hyperandrogenemia and hyperinsulinemia). Furthermore, they enable elucidation of the molecular basis of these, as well as morphometric changes in the endometrium accompanying, e.g., different types of ovarian stimulation for infertility treatment [53]. They also provide the opportunity to define molecular mechanisms predisposing to abnormal implantation and placentation resulting in, e.g., infertility, recurrent miscarriage and intrauterine fetal growth restriction. Importantly, gene expression profiling can be used to develop molecular diagnostics of endometrial normalcy and abnormalities and identifying molecular targets for therapeutic purposes in endometrial disorders.
